# Macroevolutionary bursts and constraints generate a rainbow in a clade of tropical birds

**DOI:** 10.1186/s12862-020-1577-y

**Published:** 2020-02-24

**Authors:** Jon T. Merwin, Glenn F. Seeholzer, Brian Tilston Smith

**Affiliations:** 1 Department of Ornithology, American Museum of Natural History, Central Park West at 79th Street, New York, NY 10024 USA; 2 Department of Ecology, Evolution and Environmental Biology, Columbia University, New York, NY 10027 USA

**Keywords:** Macroevolution, Bird, Color, Phylogeny, Model adequacy, Lorikeet, Mosaic evolution

## Abstract

**Background:**

Bird plumage exhibits a diversity of colors that serve functional roles ranging from signaling to camouflage and thermoregulation. However, birds must maintain a balance between evolving colorful signals to attract mates, minimizing conspicuousness to predators, and optimizing adaptation to climate conditions. Examining plumage color macroevolution provides a framework for understanding this dynamic interplay over phylogenetic scales. Plumage evolution due to a single overarching process, such as selection, may generate the same macroevolutionary pattern of color variation across all body regions. In contrast, independent processes may partition plumage and produce region-specific patterns. To test these alternative scenarios, we collected color data from museum specimens of an ornate clade of birds, the Australasian lorikeets, using visible-light and UV-light photography, and comparative methods. We predicted that the diversification of homologous feather regions, i.e., patches, known to be involved in sexual signaling (e.g., face) would be less constrained than patches on the back and wings, where new color states may come at the cost of crypsis. Because environmental adaptation may drive evolution towards or away from color states, we tested whether climate more strongly covaried with plumage regions under greater or weaker macroevolutionary constraint.

**Results:**

We found that alternative macroevolutionary models and varying rates best describe color evolution, a pattern consistent with our prediction that different plumage regions evolved in response to independent processes. Modeling plumage regions independently, in functional groups, and all together showed that patches with similar macroevolutionary models clustered together into distinct regions (e.g., head, wing, belly), which suggests that plumage does not evolve as a single trait in this group. Wing patches, which were conserved on a macroevolutionary scale, covaried with climate more strongly than plumage regions (e.g., head), which diversified in a burst.

**Conclusions:**

Overall, our results support the hypothesis that the extraordinary color diversity in the lorikeets was generated by a mosaic of evolutionary processes acting on plumage region subsets. Partitioning of plumage regions in different parts of the body provides a mechanism that allows birds to evolve bright colors for signaling and remain hidden from predators or adapt to local climatic conditions.

## Background

Animals and plants express a dazzling range of colors. Color has a direct impact on fitness through signaling [1–5], camouflage [2–4], and thermoregulation [6–8], and is a key signal of adaptive diversification and constraint. For birds in particular, plumage color plays a key role in many aspects of their diverse life histories, with notable evolutionary consequences. The major factors which drive the evolution of plumage color are climatic adaptation, crypsis, and sexual selection [4, 9]. The latter is the predominant explanation for the evolution of extreme ornamentation and colorfulness seen in various groups of birds [4, 10]. Examining the macroevolutionary trends of plumage within brightly colored clades provides a framework for understanding how natural and sexual selection interact over phylogenetic scales [9].

Typical avian clades with ornamental traits show extreme sexual dimorphism, in which males exhibit exaggerated features in form and color as compared to females, which generally have mottled brown or gray cryptic coloration [11]. In contrast, the brightly colored parrots (Order: Psittaciformes) are among the gaudiest of birds but are predominantly monomorphic [12]. As opposed to colorful dichromatic groups such as the birds of paradise (Order: Passeriformes), there is little direct evidence that any one factor such as strong sexual selection drives parrot plumage evolution, although some work suggests that assortative mating has driven color evolution in Burrowing Parrots [13, 14]. While colorful feathers may appear maladaptively conspicuous, parrot feather pigments have been linked to antibacterial resistance, solar radiation protection, and anti-predator defense [15–17]. While the characteristic bright green displayed by most parrots is decidedly cryptic against a leafy background [15–17], it is unclear whether sexual selection or drift alone have generated and partitioned the rest of the color gamut in Psittaciformes. Phylogenetic relationships among major groups of parrots are reasonably well known [18], yet few subclades have the dense taxon-sampling necessary for detailed comparative analysis. The one exception is the the brush-tongued parrots, or lories and lorikeets (Tribe: Loriini; hereafter lorikeets) [19]. Lorikeets have radiated into over 100 taxa across the Australasian region [12] since their origin in the mid-Miocene [20]. In comparison to other groups of parrots, lorikeets are species-rich given their age [20]. Their rapid diversification was likely driven by allopatric speciation as they dispersed across Australasia and may be linked to the evolution of their specialized nectarivorous diet [21]. Lorikeets have evolved an extraordinary spectrum of plumage colors that ranges from vibrant ultraviolet blue to deep crimson and black. These colors are organized in discrete plumage regions or patches which in turn vary in size, color, and placement among taxa yet are nonetheless easily defined and compared across species.

The macroevolutionary patterns that underlie the radiation of these color patches in lorikeets can provide context into how diverse coloration evolves. As with many complex multivariate traits (e.g., [22–24]), we expect that mosaic evolution, wherein subsets of traits evolve independently of others, underlies bird plumage color diversification. Different color metrics (e.g., hue vs. brightness) may be under independent selective pressures to balance a tradeoff between eye-catching ornamentation and cryptic background matching [16, 25]. For example, in the Eclectus parrot (*Eclectus roratus*) the males have bright green plumage for camouflage against predators while foraging and moving between mates, and the females have bright red and purple coloration to advertise nest-sites [16]. In the predominantly monomorphic lorikeet taxa, however, color variation appears to be partitioned along a dorso-ventral axis with the head, breast and abdominal feather regions being more variable than the wings and back. If the level of color variation is linked to whether the plumage region was subject to natural or sexual selection, or drift then distinct macroevolutionary patterns should be observable among patches. While assessing the relative fit of different macroevolutionary models cannot ascribe process, comparing their likelihoods will determine whether the distribution of color among taxa and plumage regions is consistent with a model of mosaic evolution.

In this study we quantified and modeled color evolution in the lorikeets to test whether plumage in this group is evolving as a mosaic or as a simple trait evolving under a similar evolutionary rate on all body regions. To produce color data, we imaged museum specimens, extracted spectral reflectance from plumage regions, and summarized color hue, disparity, and volume. We tested whether dorso-ventral partitioning of plumage regions can explain color evolution in lorikeets by fitting alternative evolutionary models using comparative phylogenetic methods. We predict that the relatively low color variation of dorsal plumage regions has been structured by natural selection for crypsis, and should be best explained by a model where there is a cost to evolving to new color states. In contrast, if the variable face and ventral patches are involved in conspecific recognition (sexual and social selection) they would radiate under lower constraint because there are more ways to be recognizable and visible than there are to be cryptic. Alternatively, if plumage has evolved due to a single overarching process, selection or drift might dictate the evolutionary trajectory of color variation for all patches simultaneously. Under this type of scenario, we would expect all patches to be explainable by the same model under similar parameters. Because color is often correlated with environmental conditions [26–29], we interpreted our modeling selection results in the context of the relationship between plumage color and climatic variables. Characterizing the extreme diversity of colors in the lorikeets and testing alternative scenarios that could give rise to this variation will help clarify whether discrete macroevolutionary patterns have partitioned color diversification or whether a single model will best explain the color variation in all color patches.

## Results

### Macroevolutionary model selection

**Fig. 1:**
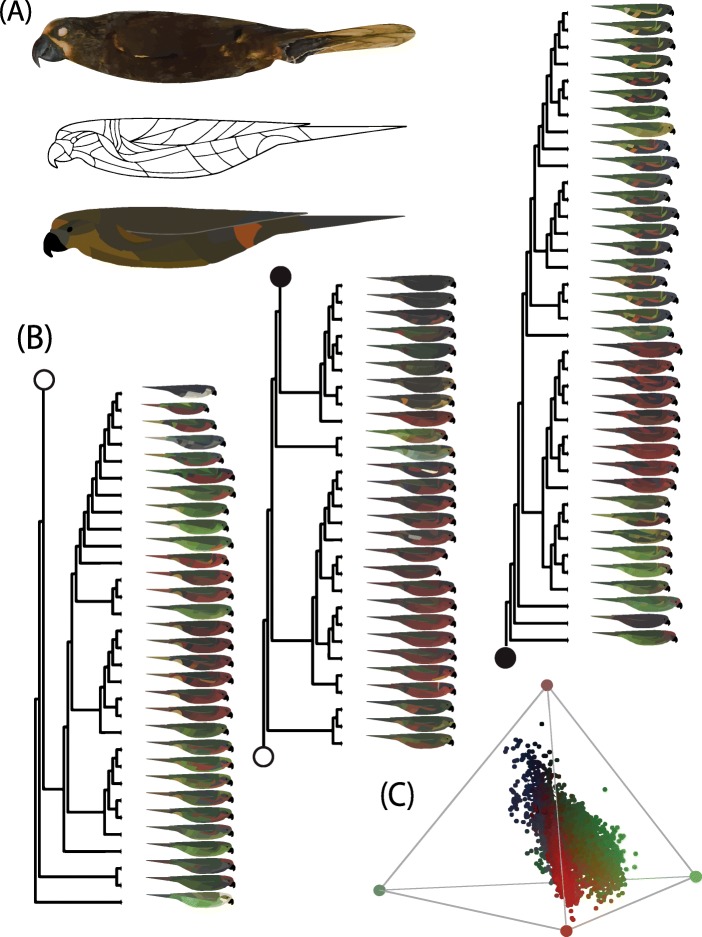
Quantifying and plotting plumage color on a phylogeny of lories and lorikeets. **a** An image of a museum specimen of *Chalcopsitta duivenbodei* (top), a blank patchmap showing the 35 plumage regions measured from images of museum specimens (middle), and the corresponding patchmap for this exemplar taxon (bottom). **b** Patchmaps of all taxa (*n* = 98) plotted on a phylogeny. The tree was split into three sections and the connecting portions are indicated with corresponding filled or empty points. **c** The tetrahedral color space of the Loriini, which contains four vertices for the four measured reflectance wavelengths: UV (purple, top), short (blue, left), medium (green/yellow, right), and long (orange/red, center). Each point represents one of the 35 color patch measurements for each taxon. The color space was centered slightly towards the longwave (red) vertex of the tetrahedral color space. While the distribution of colors in the color space skews towards the longwave part of the spectrum, it was most variant in the UV spectrum and also exhibits wide variance in the medium-wave spectrum. Colors represent the RGB colors which were mapped onto the real-color patchmaps

**Fig. 2:**
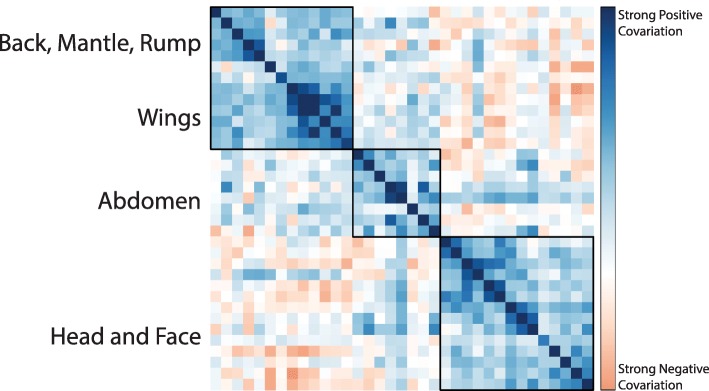
Phylogenetic variance-covariance matrix across all 35 patches shows that patch color in discrete morphological regions covary. Darker blue colors represent stronger positive covariance while darker red colors represent stronger negative covariance. Boxes represent hierarchical clusters, estimated using the built-in hclust method in corrplot

**Fig. 3:**
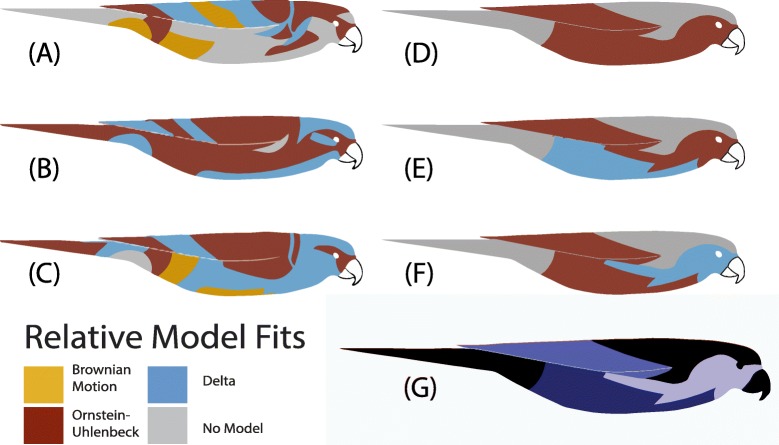
Relative model fits show a mosaic of best-fit models across patches for PC1-PC3 (**a-c**), and that most patches were a good absolute fit to the data. Different colors represent different evolutionary models and only patches with good absolute fits were plotted. The three maximum-likelihood covariant patch regions (modules) are shown in (**g**). Macroevolutionary models were fit to each module for PC1 (**d**), PC2 (**e**), and PC3 (**f**). Note that no patches or modules were best fit by White Noise models

**Fig. 4:**
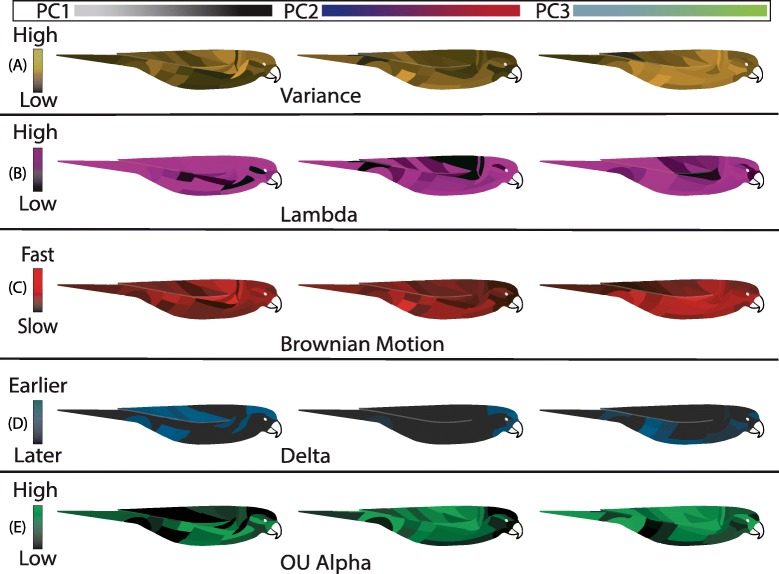
Patchmaps show within and among patch variability in PC values, model parameters, and best-fit models. PC scale bars at top show axes of color variance encompassed by each PC. Each patch in a patchmap was colored according to values for principal component variance (**a**), the modeled parameters lambda (**b**), Brownian Motion rate (**c**), delta (**d**) and OU alpha (**e**), and the best-fit model, after model adequacy (**f**). The left and right patchmaps within each panel represent PC1 and PC2, respectively. From top to bottom, the darker patches are less variable across taxa (**a**), have less phylogenetic signal (**b**), are evolving slower (**c**), diversified closer to the tips of the tree (**d**), or were relatively more constrained (**e**). See Additional file 4: Table S2 for a full listing of model-fit parameters

We found that independent patterns and rates have indeed generated color variation in the lorikeets (Fig. 1), but our results were more nuanced than our proposed alternative scenarios (Fig. 2). One extra level of complexity was that best-fit models for individual patches varied among principal component (PC) axes (Figs. 3 and 4). The first principal component (PC1, representing 52% of variance) of color primarily represented brightness, while the second (PC2, 27%) and third principal components (PC3, 13%) represented hue in the blue-to-red and UV-to-Green axes, respectively (Additional file 1: Figure S3). We visualized color data and model output using a 2D schematic of an outline of a generic lorikeet, hereafter referred to as a “patchmap” (Fig. 1a).

We compared the fit of four macroevolutionary models: white noise, Brownian Motion (BM), Ornstein-Uhlenbeck (OU), and Delta. A white noise process describes unconstrained trait evolution that is independent of phylogeny. BM is random trait evolution constrained by phylogeny. OU is random trait evolution constrained by phylogeny**,** but with an added parameter that pulls towards an optimum trait value. A Delta model is a Brownian Motion model but with an added parameter to model a rate shift.

Multi-trait, non-independent model fitting on all 35 patches showed that the highest-likelihood multi-trait model was an OU model, which is consistent with the hypothesis that all patches evolved within a constrained trait space. However, the variance-covariance matrix of this model fit showed hierarchical clustering of covariant patches on the head, abdomen, and wing (Fig. 2). When we tested alternative scenarios of trait grouping, we found that three separately evolving modules on the face, breast, and wing was the maximum likelihood scenario (Fig. 3d). Multi-trait model fitting on only these correlated patch subsets indicated that head and breast patch chromatic variation was best explained by a late-burst Delta model, while for wing and abdominal patch chromatic variation an OU model was recovered. For achromatic variation (brightness) an OU model best fit all patch regions.

We next found the best-fit models of individual patches within the covariant modules. (Fig. 2). To better understand the best-fit models for each patch, we plotted the variance, phylogenetic signal, and model fit parameters for each patch (Fig. 4). Consistent with the multi-trait model fit, breast and face patches were best fit to Ornstein-Uhlenbeck (OU) models. In contrast, in PC1 (achromatic variation or brightness), late-burst Delta and Brownian Motion models were best fit to the dorsal patches of the wings, back, and crown. Hue principle components showed the opposite pattern. PC2 (blue-to-red chromatic variation) for the forehead, crown, and occiput was best fit by Brownian Motion models. Lambda parameter (λ) values for these patches was one, indicating a rate of evolution equal to the expected signal under a random walk along the phylogeny. Face, breast, and tail evolution was best supported by a late-burst Delta (δ) model. All other patches for PC2 were best supported by an OU model. The best-fit model for most patches was selected with high relative support by sample-size corrected Akaike Information Criterion, (ΔAIC_C_ > 4) except for crown, forehead and occiput (Δ AIC_C_ < 2; Additional file 1: Figure S1). For PC2, wing, wrist, rump, and breast were best fit by an OU model. The best-fit model of PC2 for lower abdominal patches, lateral neck, and tail was Brownian Motion, while an OU model explained half of the wing, wrist, eyeline, and lower breast color. All other patches, which were clustered around the abdomen, head, and face, were best modeled by a late-burst Delta model. We found that most best-fit models were a good absolute fit to the patch color data (Fig. 3f). Undescribed rate heterogeneity for the face and breast (PC1) caused model-adequacy to fail for these patches. When we assessed model adequacy by comparing statistics estimated from empirical and simulated trait values, we used a four-statistic threshold for determining absolute fit, but many patches would have passed a five- or six-statistic threshold (Additional file 3: Table S1). Most best-fit models were robust to simulation tests and our absolute adequacy filter in arbutus (Additional file 4: Table S2) [30]. Of the six calculated model adequacy statistics, C_*var*_, the coefficient of variation of the paired differences between the estimated node and tip values, most frequently deviated from empirical values (Additional file 4: Table S2).

We examined parameter values for all models and all individual patches to understand the evolutionary dynamics between and within covariant patch regions. Overall, lambda parameter values (λ) were high, suggesting that color of even individual patches is a strong signal of phylogenetic relatedness. An examination of the parameters estimated from all tested models shows how phylogenetic signal (the extent to which phylogeny explains trait variation) varied among patches and hue and brightness. For all principal components and for most patches, fitted λ values were at the upper bound of the metric, indicating that phylogenetic signal was equal to the expected signal under Brownian Motion (Fig. 4b, Additional file 4: Table S2). For color PC1 (brightness), the malar region, and patches along the side (side breast) had the lowest phylogenetic signal (Fig. 4b). In contrast, the back, wrist, and crissal patches exhibited the lowest phylogenetic signal for color PC2 and PC3. Fitting BM models to all patches and comparing sigma parameter values showed that the fastest rate of evolution of PC1 was in the back, wrist, and abdominal patches (Fig. 4c). When we fit a Delta model to all patches we found that all patches fit models with δ > 1, indicating a late-burst pattern of evolution across patches (Fig. 4d). For PC2, many model δ values were at the default maximum, 3. For PC3, δ was 3 for the wings, body, crown and crissum, but lower on the tail, back and side-throat. As opposed to δ values, OU alpha (α) values differed greatly between patch regions. High α values, which represent stronger pull towards an estimated optimum, were fit to lower abdomen patches, wings, and wrists. The areas under weakest constraint (low α) were the breast, face, and head patches.

### Ancestral reconstruction and body size

To compare the evolution of color to a proxy for body size, we compared continuous color mapping of single patches versus wing chord length (Additional file 1: Figure S2). On face patches, we found repeated evolution of patch colors across distantly related genera and high color divergence between closely related genera. However, wing chord length was largely conserved within genera (Additional file 1: Figure S2).

**Fig. 5:**
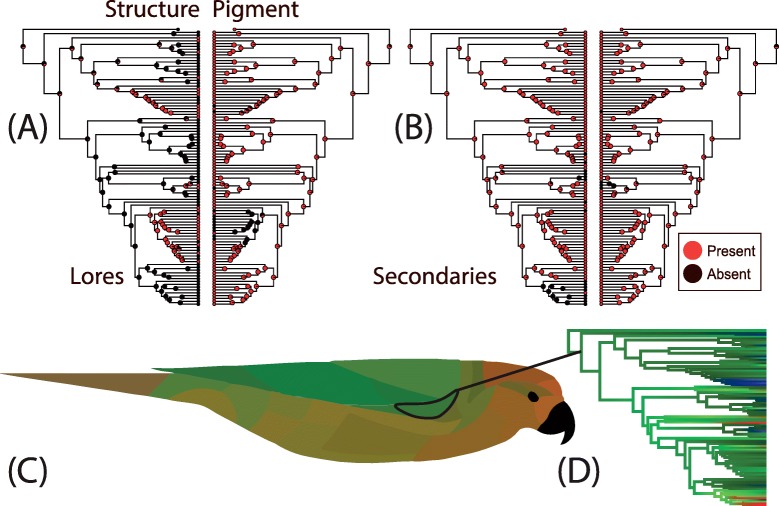
Visualization of ancestral reconstruction of plumage mechanism and color shows conservation of wing color as green and greater variability in face and breast patches. Top row represents ancestral reconstructions of plumage mechanism for two exemplar patches, the Lores (**a**) and the Secondaries (**b**). Overall, pigment was usually present but structural color fluctuated. **c** represents a continuous character ancestral reconstruction of color for all patches at the basal node to all lorikeets. Note the lighter-reddish-colored underside countershading to the dark green dorsal wings and back, which are conserved across deep nodes and found in most extant lorikeets. Ancestral states were estimated using models from the grouped patch analysis. **d** represents an example of continuous ancestral reconstruction of a single patch (the lesser coverts) with an arrow pointing to the node from which ancestral states were extracted for each patch. While the colors in (**d**) were approximated based on a single PC axis, the ancestral colors reported in (**c**) were calculated based on RGB and UV reflectance simultaneously

We compared relative gains and losses of plumage color mechanisms in each patch and found that structural color appeared and disappeared much more frequently than pigment across the evolutionary history of lorikeets (Fig. 5a-b). This pattern was much more pronounced on face patches versus wing patches. Across all patches, pigment was estimated to be present at early nodes with high likelihood. On wing patches, both pigments and structural colors were likely present at deep nodes on wing patches. For face patches however, the presence of structural color at deep nodes was uncertain.

We constructed a patchmap of mean ancestral color states of all patches using the anc.ML method in phytools and our best-fit models for each patch [31]. Patch colors on the face and abdomen change from node to node, while similar wing colors (mainly green) are distributed across the tree and are generally conserved between nodes (Fig. 5b, Additional file 1: Figure S2). The resulting ancestral lorikeet had dark green wings, a lighter red-green torso, a red crown and forehead, and purple cheek patches, closely resembling aspects of both *Trichoglossus chlorolepidotus* and *Charmosyna rubronotata* (Fig. 5a). Ancestral patchmaps plotted using the maximum or minimum of the 95% CI were qualitatively similar to those made with the mean ancestral color value and were not plotted. To further visualize how color evolved across the tree, an animation of ancestral state patchmaps from the root of the lorikeets to the basal node of *Lorius lory lory* (Additional file 1) is provided as an exemplar.

Ancestral reconstruction is a controversial analysis, and reconstruction under inaccurate or inappropriate models or even among alternative methods can often yield divergent results [32–34]. However, as per recommendations in the literature [35, 36], we performed ancestral reconstruction after a robust model selection process and compared ancestral plumage color estimates to plumage color of extant taxa. We therefore advise cautious interpretation of our ancestral reconstruction. The reconstruction of the wings as light green is realistic, and is a color found among many extant lorikeets while the reconstruction of the abdomen was a ruddy-brown halfway point between green and red which is not found in any extant lorikeets. Because green-to-red transitions are discrete (facilitated by a loss of structural color, Fig. 5a-b) we warrant caution against direct interpretation of these abdominal colors as representative. However, the overall results suggest that green dorsal plumage was ancestral and has been conserved while ventral patches have varied more frequently between red and green.

### Color and climate

Lorikeets occupied 33.5% of the colors predicted to be perceivable by tetrachromatic birds. The average color volume per taxon was 0.00513, which represents a relative volume of around 2.37% (median 2.07%) of the total avian visual space. Individual taxon color volumes ranged between 0.04 to 11.7% of avian visual space. The average hue angle variance between two patches for one bird, also known as hue disparity, was 0.912 (median: 0.962) which indicates that on average, most measured taxa displayed multiple colors from disparate areas of color space [37].

Overall, wing and abdominal color was darker and greener in warm wet areas (consistent with Gloger’s rule), but we found no such relationship between face color and climate. A phylogenetic generalized least-squares (PGLS) analysis modeling the relationship between climate and color found nuanced patterns that showed that color was correlated with precipitation, temperature, and elevation, but the nature of these relationships varied between modeling all patches at once or patches in distinct patch regions. We report multivariate models that were selected using AIC values, which were assessed as we sequentially removed insignificant model variables (with highest *p*-values) until the AIC of the new model was not significantly statistically different than the previous model, in which case the previous model was selected (ΔAIC < 2). All models had the same number of response variables (*n* = 91). Full variable loadings for each climate principal component are available in the supplementary material (Additional file 1: Figure S7).

When we modeled all patches as a response variable and principal components of climate as predictors, brighter feathers (lower color PC1) were weakly correlated with warmer (Temperature PC2) and drier (Precipitation PC2) environments (*R*^2^ = 0.05; *p* < 0.05; Table 2). Climate did not explain a significant portion of blue-to-red variation (color PC2). Wing color was more correlated with climate than face or abdomen when we correlated small groups of patches with climate (as opposed to modeling the color of all patches at once). The strongest relationship we found was on the wings, where greener color was associated with higher seasonality and higher temperatures (*R*^2^ = 0.12; *p* < 0.01). On the abdomen, we found that darker plumage was associated with lower temperatures, higher precipitation seasonality, and lower elevation (R^2^ = 0.05; *p* < 0.05). Wing patch hue PC1 (*R*^2^ < 0.01; *p* = 0.42), abdomen hue (R^2^ = 0.04; *p* = 0.10), and face patches overall (R^2^ = 0.001; *p* = 0.3) were poorly predicted by climate or elevation. Overall, those patches which were best explained by an OU model covaried most strongly with climate.

## Discussion

The evolution of the exceptional color variation in lorikeets was best explained by independent patterns or rates acting on different plumage regions and axes, namely brightness and hue. Overall, both independent patch and correlated patch subset analysis showed that while some plumage regions were drawn towards optimum values over time, others diversified along the phylogeny in bursts, suggesting that different plumage regions are subject to alternative evolutionary regimes. As is the case with many traits that characterize color [4], the plumage of lorikeets was only partially explained by climatic variation, although that variation did follow Gloger’s rule (darker in high-moisture areas). Patches that covaried with temperature and/or precipitation were conserved across the phylogeny. In contrast, those patches that evolved in bursts and were not associated with climatic variation may be evolving in response to sexual selection or due to drift. Collectively, our results suggest that at a phylogenetic scale, lorikeet plumage color has evolved in correlated regions, a pattern consistent with the idea that natural and sexual selection independently acted on components of a multivariate phenotype.

### Functional underpinnings of mosaic evolution

We found that plumage evolution has been partitioned between the back and the front (dorso-ventral axis) and between the face and the rest of the body in the lorikeets, indicating that the patterns that govern plumage evolution vary with regard to location on the body of an organism. Selected best-fit models clustered in independent units on the face, breast, and wing; and these regions are readily interpretable based on our functional knowledge of plumage color biology. For instance, the crown, forehead, and lower abdomen were best supported by a late-burst Delta model, but were poorly predicted by climate. Behavioral data suggests that these regions may be under sexual or social selection. One taxon in our dataset, *Trichoglossus haematodus* is known to flare and preen their bright crown and forehead feathers during courtship [38], but any specific role color plays in this display is not well known. An OU model fit to hue for most wing and body patches is consistent with either a constraint on evolution to new hue states for climatic adaptation, or cryptic background matching. In the forest canopy, green body and wing color may serve the purpose of camouflage against predation [6, 39, 55], while brighter plumage colors may serve as signals, as observed in the reversed sexually dichromatic parrot *Eclectus roratus* [16]. Highly variable and colorful regions, like the face, breast, and tail, were best explained by a Delta model both in the individual-patch and module model fitting. Our inferred δ parameters were greater than one, which indicates color variance within these patches evolved towards the tips of the tree. Although this pattern can be interpreted as evidence for character displacement [40, 41], the majority of taxa within clades are currently allopatric [12], so recent color evolution was presumably unaffected by interactions with other lorikeet taxa. Instead, rapid bursts of evolution across many color patches likely reflects the commonly observed pattern of rapid color evolution at the tips of phylogenies, which may indicate that these patches may function as signals to conspecifics or may be under sexual selection [37, 42]. We did not measure color of females because most lorikeets do not exhibit sexual dichromatism, and we specifically wanted to understand how ornamental color evolves in monomorphic taxa. In those lorikeets that do exhibit sexual dichromatism (e.g., some taxa in *Charmosyna*), the face patches are the regions that vary in color [12].

The difference in evolutionary dynamic that we observed between lorikeet face and wing patches may be driven by divergent selective forces. Within the Loriini and across Psittaciformes, green wings are a common phenotype [12], as 90% of parrots have green patches and 85% are primarily green [43]. The fact that wing patches were best explained by an OU model may indicate there is a selective cost to evolving away from green. Species with green wings and backs are predicted to have increased camouflage in trees against aerial and terrestrial predators [16]. While we found a correlation between climatic factors and color on the wings and the abdomen, this pattern did not hold for face patches. In contrast to monochromatic birds, which may be under strong selection for uniform plumage color (such as the snow-colored winter plumage of Rock Ptarmigans) [44], lorikeet faces may be colorful, in part, because their color variation is not constrained by natural selection. Regions with high hue variation, like the breast and face, were not explained well by an OU model, indicating that there has been no “optimum” value for the hue of these patches across the radiation of lorikeets. Therefore, these small, variable facial patches and bright breast patterns present across lorikeets may be important signals to conspecifics, while monochrome green dorsal feathers may provide cover from predators against green canopy backgrounds.

The direction and magnitude of color-climate relationships differed between principal components of color and between plumage regions. Discrete body regions showed divergent association patterns between hue and climate. Across all patches, birds were brighter in seasonal, dry areas, and darker in wet areas, supporting Gloger’s rule [29, 45]. In lorikeets, brightness and hue may be subject to different forces, a pattern which has been chiefly observed in less chromatically variable birds. Overall, the strongest relationship that we found was between wing color greenness and temperature, precipitation, and elevation. Our results suggest that birds at higher elevations and in warmer temperatures had greener wings. While wing color was most correlated with climate, abdomen and face patches showed a less pronounced or no pattern, suggesting that ornamental and cryptic coloration in lorikeets are balanced along the dorso-ventral axis.

### Model adequacy

All of our best-fit models had good absolute fit. Prior work based on non-color traits found that relative models fit to subclades within a family-level phylogeny (the Furnariidae) had good absolute fit, but these same models had poor absolute fit when applied at the family scale [30, 46]. In our dataset, simulated values of one statistic (C_*var*_) frequently deviated from empirical values because of unaccounted-for rate variation in our best-fit, constant rate model. Even at relatively shallow phylogenetic scales, body size and plumage color exhibit rate heterogeneity [23, 46, 47]. Accounting for rate shifts by testing the Delta model was critical for accurately characterizing the evolution of highly variable regions, which may be rapidly shifting between several discrete states or diversifying due to sexual selection.

### Independent or correlated patches

The developmental architecture that underlies potential concerted evolution among feather regions remains unknown for most birds [48, 49]. We found that there were three clusters of correlated patches that correspond to adjacent sections on the wing, breast, and face (Additional file 1: Figure S3). We found that these clusters were correlated when hierarchically clustered in a phylogenetic variance-covariance matrix (Fig. 5) and when analyzed in a phylogenetically-naive likelihood framework against alternative clustering hypotheses (Fig. 3c). These regions may be developmentally linked, under similar selective regimes, or the result of differential regulation of separate genes across patches or patch regions [50]. Regulatory controls on feather color may work at patch-level, feather tract-level, or whole bird-level scales [48, 49, 51, 52], and understanding how these pathways are connected will elucidate how complex plumage colors and patterns evolve. For example, most lorikeets have all-green wings with black-tipped primaries, and our ancestral reconstruction analysis indicates that the ancestor to all lories had green wings, but some *Eos* taxa have evolved red wings with black barring and UV coloration on some wing patches, demonstrating a clear interplay between region- and patch-level pigment and structural color regulation. In the sister taxon to lorikeets, *Melopsittacus undulatus*, a single base-pair change expresses tryptophan, blocking expression of yellow pigment, changing the mostly-green wild-type to a pale-blue across all patches [49]. A similar simple molecular change may explain the evolution of the two brilliant blue taxa in the Loriini; *Vini ultramarina* and *V. peruviana*, or the evolution of red-colored lorikeets in the genera *Eos*, *Pseudeos*, and *Trichoglossus* [43]*.*

### Colorful groups have recurring colors

When individual clades radiate across a high percentage of the available color space, then the repeated evolution of similar colors may be a common feature [9, 53]. For example, the robust-bodied and short-tailed lorikeets in *Lorius* and the distantly related, slender and small, long-tailed lorikeets in *Charmosyna* both have red bodies and green wings. Ancestral states inferred from continuous ancestral character estimation and discrete mechanism reconstruction, while subject to a high degree of uncertainty (Fig. 5, Additional file 1: Figure S2), indicate that green wings may have been historically conserved across this radiation and red bodies have originated multiple times (Fig. 5). Despite lorikeets being exceptionally colorful, their radiation was not characterized by constant gain of new colors, but rather repeated evolution of similar colors across the phylogeny and frequent gains and losses of structural color. Novel color evolution in birds is modulated by interactions between genes, gene expression patterns, structures, and existing metabolic pathways [48, 49, 54]. Biochemical constraints likely played a role in this plumage convergence because parrot feather color is controlled via regulatory pathways as opposed to dietary pigmentation [55]. Certain trait shifts, such as loss of ancestral yellow/green pigments and gains of red, are common across parrots [43]. In lorikeets, this may be due to the observed pattern of convergent loss of structural color (Fig. 5a-b). In carotenoid-based color systems such as in the songbird genus *Icterus*, a relatively small number of color states rapidly oscillate, leading to convergence in carotenoid and melanosome-based colors [56, 57]. A similar process may be occurring in lorikeets despite the chemically unique non-dietary pigmentation found in Psittaciformes. Regardless of mechanism, architectural constraints on plumage color or morphological traits may produce similar looking but distantly-related taxa.

### Challenges in studying plumage color

Quantifying color from museum specimens presented numerous challenges. Using museum specimens instead of hand-painted plates from field guides was preferable to us because skins exhibit UV reflectance, and the three-dimensional variation of the specimen can be captured. However, the variable preparation of museum specimens may expand or obscure certain feather patches. Therefore, we relied on subjective judgement and consultation of multiple skins, plates, and photographs when creating and implementing our patch sampling ontology. Patch outlines were drawn by hand to account for preparation style. One possible solution for patch delineation could be through random sampling of patch location [58]. The potential error in our approach pertains mostly to patch delineation, not the overall color volume of the entire bird. Despite our concerns about the subjectivity in identifying the location of patches on specimens, much of the potential error was likely minimized because of the overall morphological similarity across our focal clade as well as the fact that we performed most elements of our analysis on correlated patch groups. Additionally, patchmaps and field guide plates were qualitatively similar. In studies that sample across much deeper phylogenetic scales, identifying and sampling homologous patches will be a much more complicated task. Machine learning approaches, possibly guided by evo-devo data on feather color and pattern regulation [51], may lead to more objective patch-specific analyses. Delineating high-contrast boundaries would enable patch geometry and boundaries to be objectively quantified [51, 54] and provide a clearer means of interpreting patch colors in the context of sexual or social signaling.

## Conclusion

We found that alternative macroevolutionary models clustered in three groups on the face, abdomen, and wings best explained the exceptional color variance in the lorikeets. Such mosaic evolution is consistent with the view that separate selective and stochastic processes help shape different plumage regions and have enabled lorikeets to evolve extreme colors despite the selective costs of conspicuous coloration. Demonstrating that mosaic evolution operates in birds and other animals will clarify how extreme phenotypic diversification occurred under variable evolutionary pressures.

## Methods

### Specimen imaging, color extraction, and visualization

To quantify color, we photographed the lateral, ventral, and dorsal sides of one male museum skin for 98 taxa deposited at the American Museum of Natural History (Additional file 5: Table S3). This sampling represents 92% of the described diversity in Loriini, all described genera, and all taxa for which phylogenomic data exists [19, 20]. Because the majority of lorikeets do not exhibit sexual dichromatism, we chose only males to standardize our sampling scheme [14]. Specimens were photographed using a Nikon D70s with the UV filter removed and a Novoflex 35 mm lens. All specimens were lit using four Natural LightingNaturesSunlite 30-W full spectrum fluorescent bulbs (5500 K, 93 CRI) attached to arms mounted to a metal copy stand. Using baader spectrum filters affixed to a metal slider, specimens were photographed in both “normal” Red/Green/Blue (RGB) color as well as in the UV spectrum [59].

We demarcated 35 homologous plumage patches on the images produced for each specimen to quantify the variation among taxa based on examination of specimens, plates, and plumage topography maps (Fig. 1a; Additional file 1: Figure S1). Using the multispectral imaging package (MSPEC) in ImageJ [60] we linearized images in DCRAW and normalized images to five gray standards placed alongside each bird and extracted RGB and UV reflectance for each patch. Linearization and normalization control for light balance, maximize contrast, and standardize photographs to known reflectance values, enabling the extraction of objective color data [60]. Measurements were collected using a bluetit visual model in the MSPEC program, which transformed the data from the camera-specific (Nikon D70s) color space into an objective color space and then into a tetrachromatic avian visual model supplied with MSPEC [60]. Data was plotted in tetrahedral color space using the R v. 3.4.3 [61] package pavo v. 1.3.1 [62]. Using pavo, we extracted summary statistics (volume, relative volume, hue angle, and hue angle variance) of color spaces at varying phylogenetic scales within the Loriini and generated relative hue variables which were scaled to 1 (Table 1). We also measured wing-chord and tarsus length as proxies for body size [63].

**Table 1:** Color space statistics for all sampled taxa. All statistics were calculated within a tetrahedral color space using relative reflectance for UV, Short, Medium, and Longwave reflectance. The taxon which occupied the greatest volume of color space was *Phigys solitarius* but taxa in *Trichoglossus* comprised a large portion of the 30 taxa with the greatest color volume. The taxa which occupied the smallest amount of color space were *Vini peruviana*, which is mostly blue, and several *Chalcopsitta* taxa which are monochrome black, brown, and dark red

Taxon	Centroid UV	Centroid SW	Centroid MW	Centroid LW	Color Space Volume	Color Space Relative Volume	Color Span (Mean)	Color Span (Variance)	Hue Hisparity (Mean)	Hue Disparity (Variance)	Mean Saturation	Max Saturation
*Phigys solitarius*	0.3305	0.0884	0.2565	0.3246	0.0255	0.1178	0.4018	0.0529	1.2345	0.5704	0.7334	0.9783
*Charmosyna papou papou*	0.2534	0.1410	0.2329	0.3727	0.0210	0.0969	0.2638	0.0202	1.2389	0.5251	0.4838	0.8981
*Trichoglossus forsteni mitchellii*	0.2783	0.1001	0.3315	0.2900	0.0154	0.0712	0.2704	0.0220	–	–	0.6078	0.9452
*Trichoglossus haematodus djampeanus*	0.3503	0.1298	0.2858	0.2341	0.0153	0.0706	0.3124	0.0343	–	–	0.5482	0.8586
*Trichoglossus forsteni forsteni*	0.3281	0.1246	0.2957	0.2516	0.0139	0.0644	0.2704	0.0237	–	–	0.5185	0.8342
*Charmosyna papou wahnesi*	0.3324	0.1159	0.1857	0.3660	0.0123	0.0569	0.3240	0.0287	1.1874	0.5509	0.6313	0.9801
*Trichoglossus ornatus*	0.2565	0.1008	0.3181	0.3246	0.0107	0.0495	0.2762	0.0207	1.1066	0.3850	0.6198	0.8687
*Oreopsittacus arfaki grandis*	0.2390	0.1068	0.3922	0.2621	0.0105	0.0483	0.2307	0.0298	0.9073	0.4566	0.6063	0.9573
*Charmosyna papou goliathina*	0.2325	0.1133	0.1947	0.4594	0.0101	0.0467	0.2995	0.0319	1.0874	0.6671	0.5862	0.8683
*Trichoglossus haematodus caeruleiceps*	0.3040	0.1318	0.3024	0.2618	0.0097	0.0448	0.2955	0.0330	1.2093	0.6769	0.6278	0.8734
*Oreopsittacus arfaki major*	0.2879	0.1633	0.2879	0.2609	0.0094	0.0432	0.1878	0.0132	1.1264	0.3948	0.3841	0.7122
*Charmosyna papou stellae*	0.2764	0.1145	0.1885	0.4205	0.0092	0.0427	0.3159	0.0348	1.1741	0.6715	0.6217	0.8698
*Lorius lory lory*	0.2607	0.1998	0.2255	0.3140	0.0086	0.0399	0.2137	0.0118	1.4957	0.6147	0.3329	0.6211
*Trichoglossus capistratus flavotectus*	0.3379	0.1077	0.3263	0.2281	0.0084	0.0386	0.2461	0.0213	0.9974	0.4327	0.5952	0.9458
*Trichoglossus forsteni stresemanni*	0.2842	0.1250	0.3274	0.2634	0.0082	0.0377	0.2192	0.0145	–	–	0.5104	0.8283
*Trichoglossus capistratus capistratus*	0.2699	0.1151	0.3805	0.2345	0.0078	0.0362	0.2464	0.0216	0.9752	0.4058	0.5659	0.9776
*Psitteuteles versicolor*	0.1454	0.1319	0.3641	0.3586	0.0078	0.0361	0.1682	0.0080	0.6365	0.1351	0.5652	0.8636
*Trichoglossus rubritorquis*	0.3337	0.1886	0.2401	0.2376	0.0075	0.0346	0.2657	0.0242	1.2814	0.5763	0.5488	0.8733
*Vini kuhlii*	0.3542	0.1131	0.2327	0.3000	0.0073	0.0336	0.2674	0.0220	1.0937	0.4049	0.6089	0.8456
*Trichoglossus haematodus nesophilus*	0.3771	0.1348	0.2727	0.2153	0.0072	0.0334	0.2205	0.0165	0.9676	0.3754	0.5085	0.7654
*Trichoglossus capistratus fortis*	0.3202	0.1058	0.3369	0.2371	0.0072	0.0333	0.2467	0.0207	–	–	0.5993	0.9304
*Psitteuteles goldiei*	0.2390	0.0920	0.3532	0.3158	0.0072	0.0332	0.2233	0.0254	0.9245	0.3598	0.6378	1.0000
*Trichoglossus haematodus nigrogularis*	0.3411	0.1319	0.2791	0.2479	0.0071	0.0327	0.2304	0.0158	1.0647	0.3802	0.5354	0.8588
*Charmosyna josefinae cyclopum*	0.2019	0.1015	0.2218	0.4748	0.0070	0.0322	0.2532	0.0267	0.8744	0.5319	0.5941	0.8389
*Trichoglossus haematodus intermedius*	0.3601	0.1405	0.2700	0.2295	0.0069	0.0316	0.2066	0.0142	0.9669	0.3517	0.4765	0.7834
*Charmosyna placentis placentis*	0.1619	0.1284	0.3917	0.3180	0.0068	0.0316	0.2167	0.0166	0.9359	0.5696	0.5449	0.8612
*Charmosyna margarethae*	0.2028	0.1002	0.2248	0.4722	0.0068	0.0313	0.2749	0.0338	0.9921	0.6008	0.6019	0.9574
*Trichoglossus haematodus flavicans*	0.3297	0.1275	0.2511	0.2917	0.0067	0.0309	0.2287	0.0177	1.0650	0.4762	0.5746	0.8845
*Lorius chlorocercus*	0.1873	0.1403	0.2288	0.4435	0.0066	0.0305	0.2435	0.0178	1.0810	0.7308	0.4974	0.8131
*Charmosyna placentis ornata*	0.1502	0.1388	0.3631	0.3479	0.0064	0.0297	0.2522	0.0195	1.0653	0.6334	0.5373	0.8908
*Lorius lory jobiensis*	0.2849	0.1668	0.1982	0.3501	0.0064	0.0295	0.2468	0.0187	1.2864	0.5228	0.4256	0.7257
*Charmosyna placentis subplacens*	0.2550	0.1358	0.3212	0.2880	0.0062	0.0289	0.2071	0.0172	1.0320	0.3771	0.4918	0.8136
*Charmosyna placentis intensior*	0.1544	0.1314	0.4006	0.3135	0.0062	0.0287	0.2472	0.0302	0.8935	0.4832	0.5127	0.7778
*Lorius lory cyanuchen*	0.2528	0.1787	0.2289	0.3396	0.0060	0.0278	0.2254	0.0139	1.3829	0.5738	0.3751	0.6147
*Charmosyna josefinae josefinae*	0.2473	0.0940	0.1738	0.4850	0.0060	0.0277	0.2532	0.0280	0.9082	0.6368	0.6277	0.8597
*Eos cyanogenia*	0.2984	0.1204	0.1518	0.4293	0.0060	0.0276	0.2872	0.0294	1.0047	0.5911	0.6441	1.0000
*Glossopsitta concinna*	0.2535	0.1534	0.3481	0.2450	0.0057	0.0266	0.1714	0.0153	0.9212	0.3581	0.4362	0.7237
*Lorius hypoinochrous hypoinochrous*	0.1930	0.1787	0.2280	0.4003	0.0057	0.0263	0.2014	0.0120	1.1047	0.6486	0.4033	0.8144
*Lorius lory salvadorii*	0.2801	0.1770	0.2155	0.3274	0.0056	0.0261	0.2017	0.0107	1.3450	0.5267	0.3639	0.6238
*Eos semilarvata*	0.2442	0.0847	0.1144	0.5567	0.0056	0.0260	0.2481	0.0362	–	–	0.7145	0.9789
*Eos histrio talautensis*	0.3401	0.1405	0.1197	0.3998	0.0055	0.0253	0.3674	0.0620	–	–	0.6969	0.9691
*Charmosyna pulchella rothschildi*	0.2924	0.1105	0.2087	0.3884	0.0053	0.0243	0.2295	0.0155	0.9755	0.4037	0.5652	0.7653
*Trichoglossus haematodus massena*	0.3202	0.1465	0.3026	0.2307	0.0051	0.0237	0.1717	0.0110	0.9429	0.3684	0.4318	0.6985
*Lorius hypoinochrous rosselianus*	0.3010	0.1405	0.1870	0.3715	0.0050	0.0233	0.2294	0.0132	1.1178	0.4494	0.4920	0.6994
*Eos reticulata*	0.2165	0.0814	0.1185	0.5836	0.0050	0.0230	0.2430	0.0372	–	–	0.7155	0.9881
*Trichoglossus haematodus haematodus*	0.3518	0.1558	0.2707	0.2217	0.0049	0.0227	0.1862	0.0123	0.9852	0.3598	0.4533	0.7106
*Charmosyna wilhelminae*	0.2058	0.1014	0.4250	0.2678	0.0049	0.0226	0.1428	0.0092	0.5966	0.2604	0.6089	0.8084
*Eos squamata squamata*	0.2697	0.0821	0.1274	0.5208	0.0049	0.0224	0.2607	0.0243	–	–	0.6792	0.9813
*Charmosyna placentis pallidior*	0.1699	0.1067	0.3987	0.3246	0.0048	0.0223	0.2263	0.0258	0.8673	0.5003	0.6018	0.8020
*Trichoglossus haematodus moluccanus*	0.3040	0.1771	0.2805	0.2384	0.0048	0.0222	0.1985	0.0159	1.1969	0.5387	0.4333	0.8387
*Vini australis*	0.3292	0.1259	0.2957	0.2492	0.0047	0.0216	0.2056	0.0194	0.9756	0.4464	0.5394	0.7386
*Chalcopsitta scintillata rubrifrons*	0.3134	0.1463	0.2557	0.2846	0.0046	0.0212	0.2180	0.0134	1.1215	0.2944	0.4911	0.7704
*Charmosyna pulchella pulchella*	0.1766	0.1277	0.2592	0.4365	0.0045	0.0207	0.2416	0.0209	0.9219	0.4789	0.5376	0.7431
*Pseudeos fuscata*	0.2325	0.1504	0.2462	0.3708	0.0044	0.0205	0.2095	0.0161	–	–	0.4974	0.9097
*Chalcopsitta scintillata scintillata*	0.2669	0.1682	0.2921	0.2728	0.0044	0.0203	0.1618	0.0081	1.1104	0.3511	0.3549	0.6123
*Eos histrio histrio*	0.3266	0.1256	0.1041	0.4437	0.0044	0.0202	0.3480	0.0583	0.9626	0.6474	0.7208	1.0000
*Eos bornea cyanonotha*	0.1977	0.0713	0.0949	0.6361	0.0043	0.0198	0.2210	0.0344	0.4871	0.3814	0.7633	0.9617
*Lorius hypoinochrous devittatus*	0.2711	0.1362	0.1754	0.4172	0.0043	0.0198	0.2394	0.0218	1.1044	0.5773	0.5061	0.7380
*Psittaculirostris edwardsii*	0.2598	0.1556	0.2965	0.2880	0.0042	0.0193	0.1974	0.0126	1.1525	0.3824	0.4084	0.6962
*Parvipsitta porphyrocephala*	0.2151	0.1879	0.3274	0.2696	0.0041	0.0191	0.1573	0.0088	1.0167	0.3977	0.3782	0.7623
*Lorius lory cyanuchen*	0.2229	0.1375	0.3442	0.2953	0.0040	0.0184	0.1994	0.0170	0.9452	0.3136	0.4615	0.7402
*Trichoglossus haematodus rosenbergii*	0.3978	0.1458	0.2489	0.2074	0.0039	0.0181	0.2151	0.0154	0.9605	0.3730	0.5268	0.8208
*Lorius domicella*	0.2252	0.1548	0.1957	0.4243	0.0038	0.0177	0.2230	0.0184	1.0613	0.7001	0.4154	0.7520
*Psitteuteles iris wetterensis*	0.2536	0.1078	0.3628	0.2758	0.0038	0.0175	0.1612	0.0086	0.7942	0.2681	0.5704	0.8158
*Trichoglossus haematodus deplanchii*	0.3228	0.1602	0.2868	0.2303	0.0036	0.0168	0.1668	0.0097	0.9893	0.3975	0.3944	0.5921
*Chalcopsitta scintillata chloroptera*	0.3647	0.1706	0.2697	0.1950	0.0035	0.0161	0.1796	0.0099	0.9430	0.3312	0.4192	0.6764
*Lorius lory viridicrissalis*	0.2602	0.1827	0.2211	0.3361	0.0035	0.0160	0.2086	0.0145	1.4276	0.6659	0.3437	0.6494
*Vini stepheni*	0.3603	0.1310	0.2412	0.2675	0.0034	0.0159	0.2255	0.0203	1.0225	0.4582	0.5348	0.7283
*Eos bornea bornea*	0.1540	0.0516	0.0933	0.7012	0.0033	0.0151	0.1543	0.0231	0.2855	0.2312	0.8122	0.9792
*Lorius lory erythrothorax*	0.2813	0.1744	0.2001	0.3441	0.0033	0.0151	0.2151	0.0167	1.3247	0.6278	0.3996	0.6412
*Parvipsitta pusilla*	0.2760	0.1431	0.3310	0.2499	0.0029	0.0133	0.1637	0.0119	0.9087	0.3013	0.4308	0.6764
*Melopsittacus undulatus*	0.1663	0.1436	0.3833	0.3068	0.0028	0.0131	0.1371	0.0045	0.5989	0.1524	0.5031	0.9410
*Charmosyna rubronotata rubronotata*	0.2833	0.1597	0.2990	0.2581	0.0028	0.0128	0.1614	0.0099	1.0223	0.3333	0.3960	0.5854
*Psitteuteles iris iris*	0.2292	0.1073	0.3855	0.2779	0.0025	0.0116	0.1374	0.0065	0.6900	0.2414	0.5729	0.7496
*Lorius garrulus garrulus*	0.2027	0.1352	0.1806	0.4815	0.0025	0.0115	0.1757	0.0178	0.7300	0.6442	0.4725	0.6596
*Charmosyna rubrigularis*	0.2447	0.1304	0.3823	0.2425	0.0024	0.0113	0.1579	0.0098	0.7551	0.2266	0.4904	0.7422
*Lorius garrulus flavopalliatus*	0.1973	0.1356	0.1893	0.4778	0.0023	0.0105	0.1847	0.0174	0.7740	0.5460	0.4651	0.6287
*Trichoglossus weberi*	0.2223	0.0947	0.4443	0.2387	0.0022	0.0103	0.1197	0.0040	–	–	0.6229	0.8492
*Neopsittacus pullicauda pullicauda*	0.3177	0.1230	0.2937	0.2656	0.0022	0.0103	0.1708	0.0125	0.8479	0.2930	0.5080	0.7471
*Neopsittacus musschenbroekii musschenbroekii*	0.0680	0.1260	0.4091	0.3969	0.0022	0.0101	0.1608	0.0117	0.4652	0.1217	0.7398	0.9968
*Lorius albidinucha*	0.2118	0.1521	0.2123	0.4237	0.0022	0.0100	0.2222	0.0222	1.2390	0.9766	0.4245	0.6914
*Lorius lory somu*	0.2476	0.1818	0.2119	0.3587	0.0020	0.0093	0.1797	0.0113	1.3471	0.6978	0.3183	0.6065
*Neopsittacus pullicauda alpinus*	0.3725	0.1381	0.2545	0.2350	0.0019	0.0089	0.1612	0.0159	0.7726	0.3649	0.4768	0.7517
*Charmosyna rubronotata rubronotata*	0.2579	0.1130	0.1522	0.4769	0.0018	0.0084	0.2365	0.0235	0.6781	0.3357	0.5728	0.8609
*Charmosyna meeki*	0.1643	0.1126	0.4582	0.2649	0.0018	0.0082	0.1035	0.0058	0.3686	0.1316	0.5516	0.7148
*Eos squamata obiensis*	0.2542	0.0900	0.1295	0.5263	0.0017	0.0079	0.2090	0.0170	0.5063	0.1781	0.6484	0.8696
*Vini ultramarina*	0.1840	0.2996	0.2944	0.2220	0.0017	0.0077	0.1782	0.0165	1.1568	0.8436	0.5007	0.7132
*Chalcopsitta atra insignis*	0.4197	0.1855	0.1712	0.2236	0.0016	0.0074	0.1656	0.0106	0.6393	0.2925	0.3728	0.5909
*Lorius garrulus morotaianus*	0.1740	0.1272	0.1827	0.5161	0.0016	0.0073	0.1859	0.0208	0.6618	0.5909	0.4974	0.7011
*Trichoglossus euteles*	0.2076	0.0962	0.3691	0.3270	0.0015	0.0071	0.1399	0.0075	0.6618	0.2379	0.6171	0.8128
*Trichoglossus johnstoniae*	0.2698	0.1256	0.3519	0.2528	0.0015	0.0070	0.1261	0.0046	0.7310	0.2006	0.4977	0.6513
*Trichoglossus flavoviridis flavoviridis*	0.2643	0.1078	0.3370	0.2909	0.0014	0.0063	0.1607	0.0079	0.8171	0.2382	0.5708	0.7898
*Charmosyna amabilis*	0.1591	0.1262	0.4035	0.3112	0.0013	0.0062	0.1528	0.0160	0.5654	0.2581	0.5001	0.6614
*Charmosyna toxopei*	0.2463	0.1269	0.3902	0.2366	0.0013	0.0062	0.1112	0.0034	0.5764	0.1335	0.5022	0.6756
*Trichoglossus chlorolepidotus*	0.2990	0.1567	0.3336	0.2107	0.0013	0.0059	0.1185	0.0040	0.7488	0.1832	0.4297	0.6663
*Pseudeos cardinalis*	0.1804	0.0623	0.1223	0.6349	0.0011	0.0049	0.1393	0.0097	0.2277	0.0572	0.7507	0.9424
*Charmosyna palmarum*	0.1738	0.1125	0.4573	0.2563	0.0009	0.0041	0.1087	0.0050	0.3933	0.0975	0.5512	0.7662
*Charmosyna multistriata*	0.1440	0.1271	0.4251	0.3038	0.0009	0.0041	0.1086	0.0028	0.4082	0.0733	0.5286	0.7878
*Trichoglossus flavoviridis meyeri*	0.2788	0.1189	0.3387	0.2635	0.0009	0.0040	0.1151	0.0030	0.6770	0.1116	0.5244	0.6900
*Chalcopsitta duivenbodei duivenbodei*	0.3054	0.1572	0.2463	0.2911	0.0008	0.0038	0.1230	0.0114	0.7341	0.3705	0.3721	0.8626
*Trichoglossus rubiginosus*	0.4108	0.1684	0.1625	0.2584	0.0006	0.0027	0.0925	0.0026	0.3955	0.1053	0.3814	0.5304
*Chalcopsitta atra bernsteini*	0.3807	0.2204	0.2002	0.1987	0.0005	0.0022	0.1066	0.0044	0.5215	0.3415	0.2774	0.5623
*Chalcopsitta duivenbodei syringanuchalis*	0.3702	0.1453	0.2205	0.2639	0.0003	0.0015	0.1382	0.0089	0.6673	0.2467	0.4230	0.7603
*Chalcopsitta atra atra*	0.3400	0.2263	0.2191	0.2146	0.0003	0.0014	0.0811	0.0039	0.5919	0.5292	0.2098	0.4456
*Vini peruviana*	0.4722	0.2126	0.1677	0.1475	0.0001	0.0004	0.2150	0.0294	1.0803	1.8047	0.5320	0.8396

**Table 2:** Best-fit PGLS models. Overall, wing patches were best predicted by climate, while we found no relationship between face color and biogeographic variables. Models were selected for each patch subset and each color principal component. Coefficients are presented in the order that they are listed under the “predictors” column, with the intercept value as the last coefficient

Predictors	AIC	Adj-R-Sqaured	*P*-Value	Coefficients	Patch Subset	Color PC
*Ppc1 + Alt*	219.4	0.0367	0.0719	*2.42167x + 0.00029x + −0.00835*	Wing	1
*Ppc1 + Ppc3*	279.7	−0.0032	0.4273	*2.97335x + 0.00028x + 0.00094*	Wing	2
*Tpc1 + Tpc3 + Ppc1 + Ppc3 + Alt*	236.2	0.1203	0.0068	*1.42404x + −0.00068x + 0.03842x + 0.0016x + 0.00191x + 0.01336*	Wing	3
*Ppc3 + Alt*	257.2	0.0152	0.1894	*3.60072x + −0.00101x + 0.006*	Face	1
*Ppc1 + Alt*	259.4	0.0105	0.2335	*3.16067x + −0.00024x + 0.00789*	Face	2
*Tpc3 + Alt*	269.0	−0.0053	0.4687	*2.74926x + 0.02832x + 0.00556*	Face	3
*Ppc3 + Alt*	247.2	0.0561	0.0293	*4.54306x + −0.00171x + − 0.00519*	Abdomen	1
*Ppc1 + Alt*	303.8	0.0133	0.2064	*3.93479x + −0.00054x + 0.00722*	Abdomen	2
*Tpc1 + Ppc2*	280.3	0.0257	0.1185	*2.18623x + −0.00026x + 0.00086*	Abdomen	3
*Ppc2 + Ppc3*	469.3	0.0499	0.0392	*17.80537x + −0.00178x + − 0.00575*	All	1
*Ppc1 + Ppc2*	477.6	−0.0012	0.3915	*19.72418x + −0.001x + − 0.00154*	All	2
*Tpc1 + Ppc1 + Ppc3*	505.6	0.0331	0.1161	*13.56358x + −0.00497x + 0.0089x + 0.00577*	All	3

We visualized color data and model output using an outline of a generic lorikeet, which we refer to as a “patchmap” (Fig. 1a). We wrote a custom R script to automatically color our patchmaps with raw reflectance data. Specifically, the script input raw blue, green, and red reflectance data into the RGB method in the R package grDevices version 3.4.3 to generate hex colors for each patch for each taxon [61, 64]. Images were plotted as tip labels on a phylogeny representing all of our sampled taxa [19] (Fig. 1c) using ggtree v. 1.10.4 in R [62, 65]. Patchmap image sizes were scaled to represent relative taxa sizes measured from museum skin wing lengths.

### Modeling color evolution across the Loriini tree

We modeled patch color across the phylogeny to visualize phylogenetic signal across patches, and to compare how much particular patches evolved from ancestral states. We predicted that patches linked to crypsis (e.g., wing) would maintain a similar color across the tree. In contrast, patches that are presumably involved in mate signaling (e.g., face and breast) would show greater disparity across the tree. First, we converted a non-ultrametric tree (Supplementary Fig. S11 in Smith et al. 2019) for the Loriini from into a time-calibrated tree using the program treePL [66]. To date the tree, we used a secondary calibration from [20] by specifying the age for the node separating the Loriini from their sister taxon *Melopsittacus undulatus* to a minimum and maximum age of 11 and 17 million years ago, respectively. We estimated the optimal parameter settings using the prime and thorough options, and the tree was dated using a smoothing value of 1, which was determined by cross validation. This time-calibrated tree was used in all downstream analyses and a comparison between the time-calibrated tree and the uncalibrated tree is available in the supplement (Additional file 1: Figure S6).

### Ancestral character estimation of all patches

To estimate ancestral plumage states for the four raw reflectance variables, we used the anc.ML method in phytools [31]. We visualized the estimated ancestral states on patchmaps to determine which specific colors have been conserved over time. The anc.ML method allows for the use of alternative macroevolutionary models (i.e., BM or OU) for ancestral reconstruction. We used the best-fit model for each patch to estimate ancestral states. Using multivariate ancestral character estimation along multiple axes was necessary because color is a fundamentally multivariate trait and ancestral estimates of single color parameters, such as brightness or a single principal component of hue, cannot account for covariation and independence between different color spectra. We layered images of ancestral states at sequential nodes into animated GIFs to visualize color conservation and shifts for an example sequence of nodes from the root to an exemplar tip (Additional file 2).

### Plumage mechanism analysis

We modeled expression of two color mechanisms, structural color and pigment. We first clustered all of our color data into 10 parts, and then categorized this color data based on whether each color was generated by a pigmentary or structural color. In general, we assumed that blue and UV color was generated by structural color, red and yellow by psittacofulvin pigment, and green by a combination of pigments and structural colors. We then modeled each color production mechanism as a binary trait on the phylogeny using the SimMap function in phytools [31].

### Macroevolutionary model selection and adequacy test

We tested whether plumage color evolution across patches in lorikeets was best explained by multiple or a single macroevolutionary model. Complex traits are often the product of different evolutionary processes; e.g., tetrapod cranial and postcranial skeletal morphology are subject to discrete forces associated with diet and locomotor strategy, respectively [67]. However, in some cases, a single model may best explain the evolution of a complex trait under strong natural selection, such as with cryptic coloration in female passerines [27]. To test these alternative hypotheses, we used a comparative phylogenetic method to select relative and absolute best-fit models for the first three principal components of color. We compared and selected best-fit models for all patches together, for correlated patches, and finally for each patch independently using AIC weights. To analyze color variation across all patches, we performed a principal components analysis of all 4620 color measurements with prcomp in R, using the four raw quantum catch variables (UV, short-, medium-, and long-wave) as factors (R Core Team, 2017). This flattens a four-dimensional color-space matrix into PCs that explain brightness and hue-opponent coordinates (Additional file 1: Figure S3).

Because treating individual patches separately in tests may lead to model misspecification due to independent analysis of non-independent traits [68], we fit models for multiple patches at once using the R package phylocurve [69]. This analysis allowed us to identify highly correlated groups of patches and estimate the best-fit model for all patches together and for these correlated groups. To identify the global (entire bird) best-fit model, we first compared alternate models in phylocurve for all patches simultaneously. We then visualized the phylogenetic covariance matrix generated by the all-patch phylocurve analysis. Covariance matrices were visualized using the corrplot package [50]. From this covariance matrix we identified correlated patch regions by comparing our proposed clustering hypothesis to null clustering hypotheses using EMMLi (e.g., patches on the wing), which finds the maximum likelihood clustering scenario among traits [70]. We then re-ran phylocurve on these patch clusters, for BM, OU, delta, and white noise models, selecting best-fit models based on AIC.

For each individual patch, we modeled PC1, PC2, and PC3 for each patch with Brownian Motion, OU, Delta, and White Noise across the phylogeny with the fitContinuous method in the geiger package in R [71] (version 2.0.6). From these models, for each patch we extracted the fit parameters Brownian Motion rate, delta rate-change (δ), phylogenetic signal (λ), and OU bounding effect (α) to assess how parameter values varied across patches independent of their best-fit model. To identify the relative best-fit model for each patch, we considered ΔAIC_C_ greater than 2 to be significantly different. These models were used to test the following expectations: colors evolved as a random walk along phylogeny (Brownian Motion), color evolved within selective constraints (OU), color evolved in a random pattern, irrespective of phylogeny (White Noise), or color evolved in late or early burst fashion (Delta).

Though model selection based on AIC_C_ identifies the best relative model, the model may be overfit and unrealistic [30]. To test model fit adequacy, we compared our empirical trait values to simulated trait values using the arbutus package [30](version 0.1). Based on a fitted model, arbutus creates a unit tree (a tree with uniform branch lengths of 1), simulates posterior distributions, and compares those simulated distributions of six statistics (Additional file 4: Table S2) to the empirical trait distribution. When simulated values differed from empirical values (two-tailed *P*-value; alpha = 0.05), the model had poor absolute fit. We then filtered out the models which failed two or more tests [30, 46]. The best-fit model for each patch was plotted on the patchmap. For patches with ΔAIC_C_ scores of < 2 among top models, the model with fewer parameters was selected. For models with identical complexity, Mahalanobis distance to simulated trait means was used as a post-hoc test in order to pick best-fit models [30, 69].

### Testing for climatic correlates with color

To test if plumage variation covaried with ecogeographical gradients, we examined the relationship between temperature, precipitation, elevation, and patch color. Although some aspects of lorikeet color, as with other ornamental clades (e.g., [25]), may not strongly covary with climate gradients, we aimed to test whether this decoupling of climate and color was present when we tested individual patch regions. Overall, we expected to find that regions involved in climatic adaptation or crypsis (e.g., wings) covary with climatic variables more strongly than regions potentially involved in signaling (e.g., face).

We used the extract function in the raster package in R to extract the median value from each of 19 bioclim variables [72] as well as elevation [73] from the shapefiles representing each taxon’s distribution [74]. Median bioclim and elevation values were calculated from all raster cells (1-km resolution) within each taxon distribution shapefile due to a paucity of accurate occurrence records for many lorikeets. We then used the PGLS method in the R package caper [75] to test the relationship between each PC axis for three groups of patches (wing, abdomen, and face) as well as all patches at once, using elevation and the first three principal components of temperature and precipitation as predictors while accounting for phylogeny and using maximum likelihood estimates for lambda. All PGLS models had the same sample size (*n* = 91), which was a slight reduction from our total taxon list because we excluded several subspecies without range data. We selected the best model for each patch group using AIC values, which were assessed as we sequentially removed insignificant model variables (with highest *p* = values) until AIC of the new model was not 2 lower than the previous model AIC (variables with the highest *p*-value).

## Supplementary information

**Additional file 1:** Contains Supplementary Figs. S1-S7 as referenced in text.

**Additional file 2: **Electronic Supplement: Root to Lorius Animation. An animation between ancestral reconstructions at each node from root of all lorikeets to *Lorius*.

**Additional file 3: Table S1.** Arbutus Statistics By Patch. Absolute fit for each of the six statistics calculated by arbutus. If the value is for a given statistic is below *p* < .05, that statistic does not support the tested model. Presented for each patch and PC.

**Additional file 4: Table S2.** Model Fit Parameters. Statistics for each of the four relative fit models (Brownian Motion, OU, Delta), phylogenetic signal, and overall variance for each patch and each color PC.

**Additional file 5: Table S3.** Modularity Maximum Likelihoods. Maximum likelihood results of EMMLi model selection.

**Additional file 6: Table S4.** Sampling Table. Table of AMNH specimens from which color data were collected.

## Data Availability

The datasets generated and/or analyzed during the current study, as well as a list of specimens used in the color quantification are available in the GitHub repository, https://github.com/jtmerwin/LorikeetColor
