# Supplementary material for: Macroevolutionary bursts and constraints generate a rainbow in a clade of tropical birds
Source: BMC Evol Biol. 2020 Feb 24;20:32. doi: 10.1186/s12862-020-1577-y (PMC7041239; doi:10.1186/s12862-020-1577-y)
Supplement: Supplementary file 1 — Additional file 1: Contains Supplementary Figs. S1-S7 as referenced in text. [file 12862_2020_1577_MOESM1_ESM.docx]

**Supplemental Material**


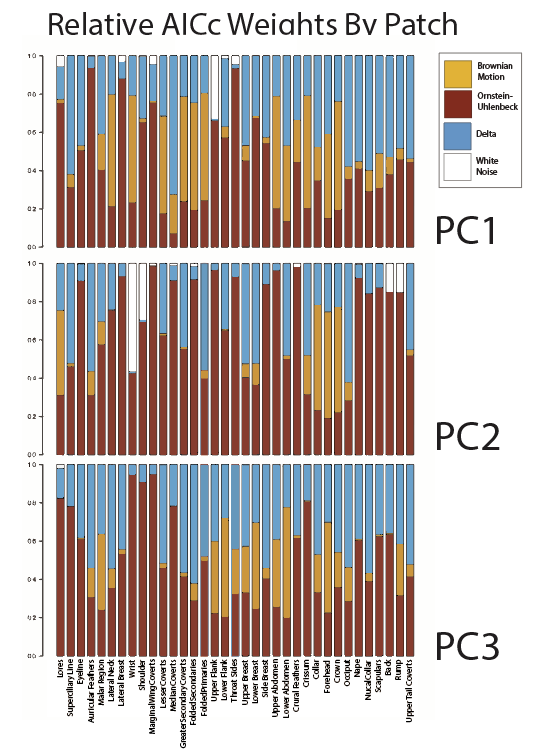


Figure S1: Relative AICc weights by patch. Note that in PC1, OU patches were selected with very high relative support.


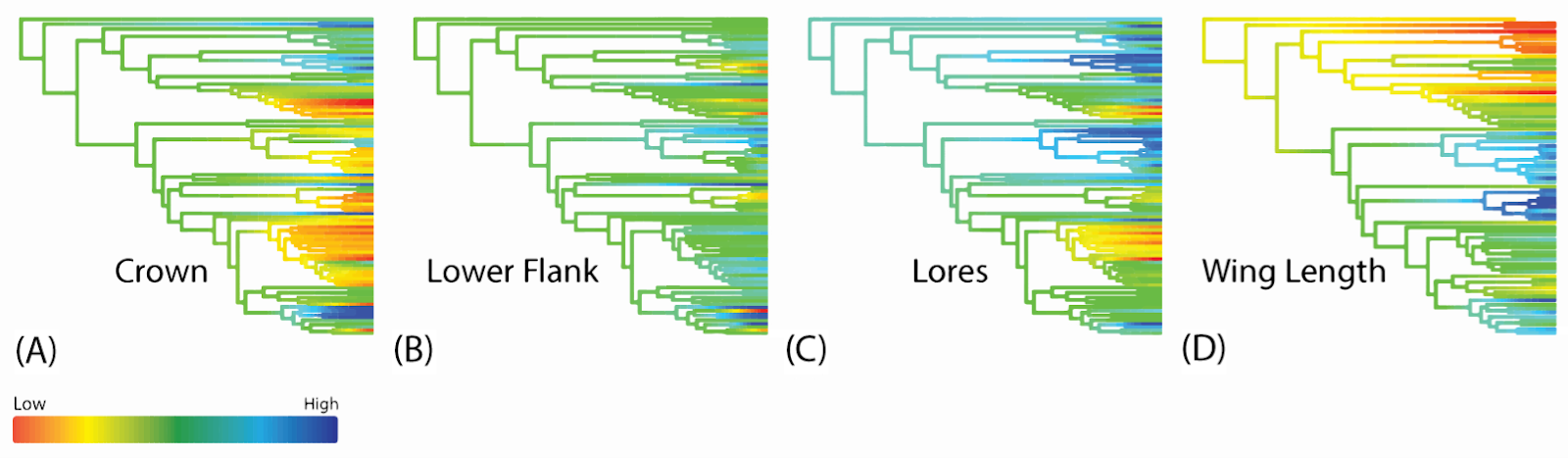


Figure S2: Continuous character mapping shows that color is a more labile trait than body size. Ancestral states were estimated using a Brownian Motion process. Included are exemplar 3 patches and wing length mapped on the phylogeny. The patches correspond to PC1 for the top of the head (A, Crown), lateral view above the legs (B, Lower Flank), area directly next to the bill (C, Lores), and a proxy for body size (D, Wing Length).


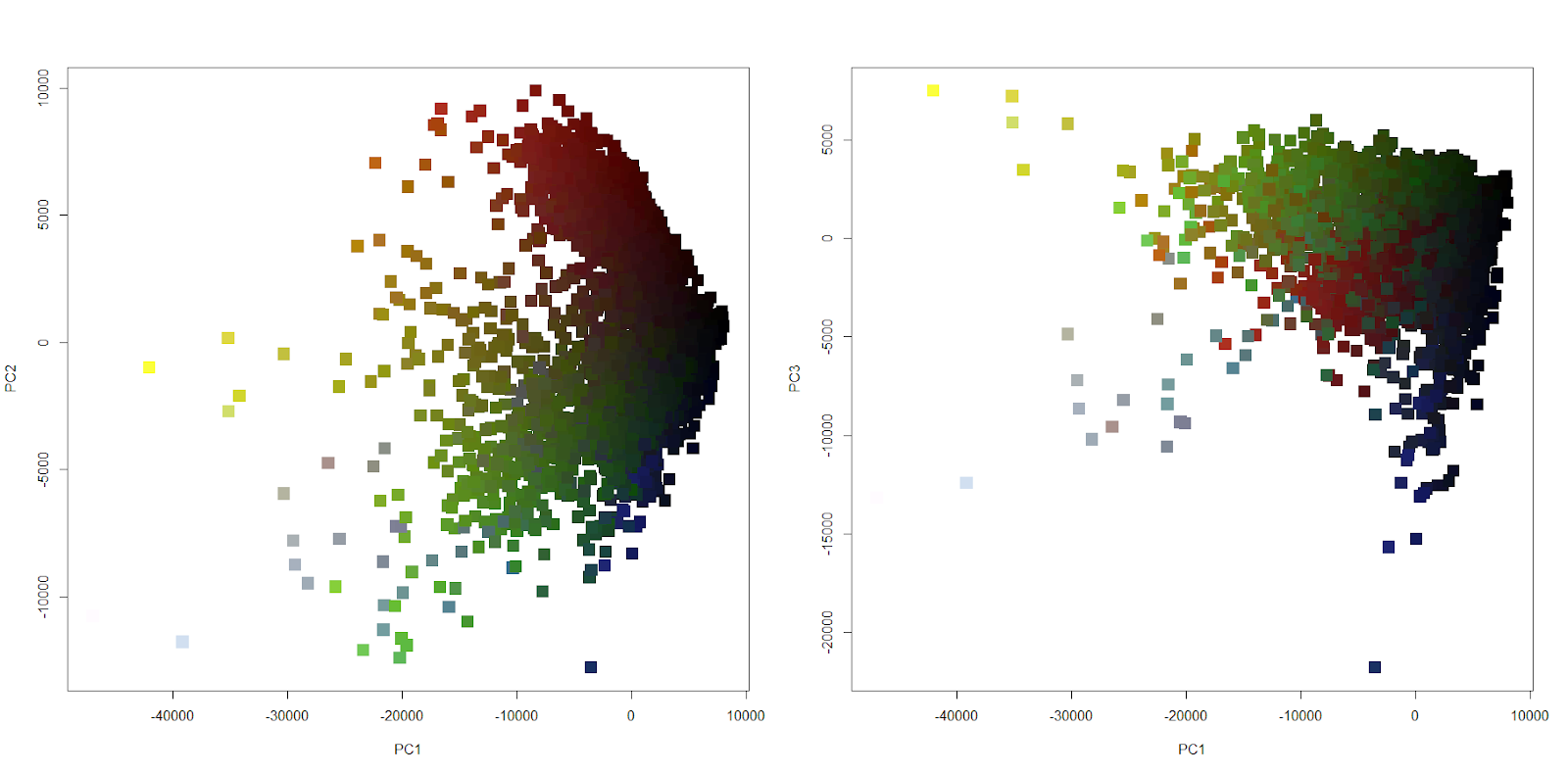


Figure S3: Scatterplot of PC1 and PC2 of Color. Color represents real colors that a human would see, as generated by the RGB method in R. This shows that a PCA across all 4,620 color measurements using the four reflectance variables (U, S, M, and L) as factors separates into achromatic brightness on PC1 and hue, running from shortwave to long-wave on PC2.


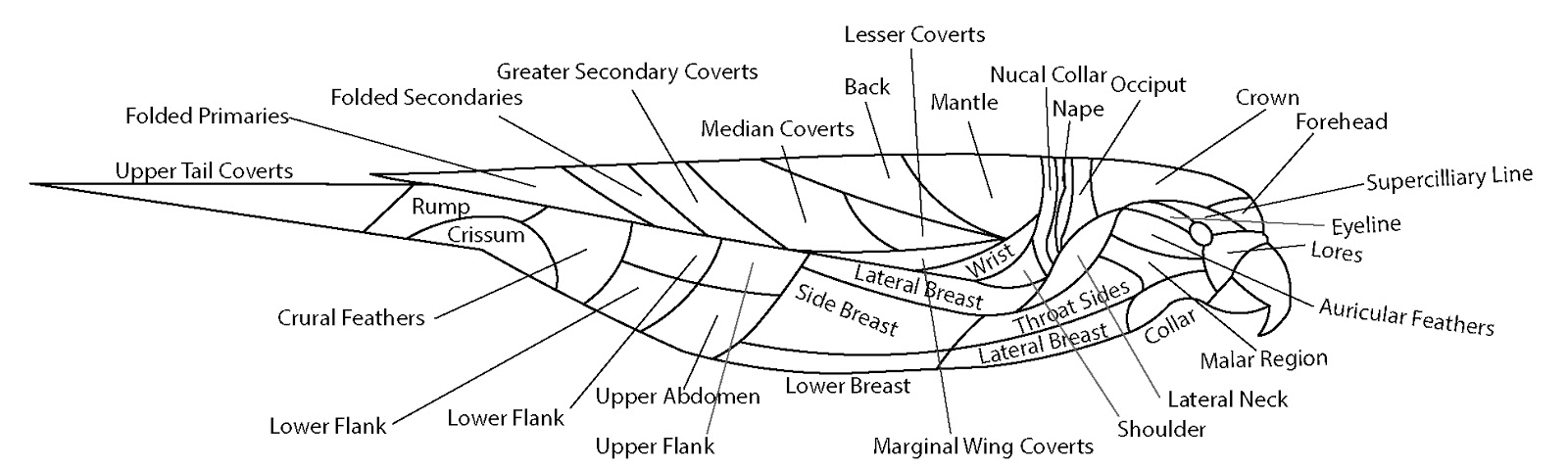


Figure S4: Patchmap showed labeled plumage regions. Color was extracted from these 35 regions on each specimen. The position of patches on specimens was visually approximated and hand delineated on images as per figure S5.


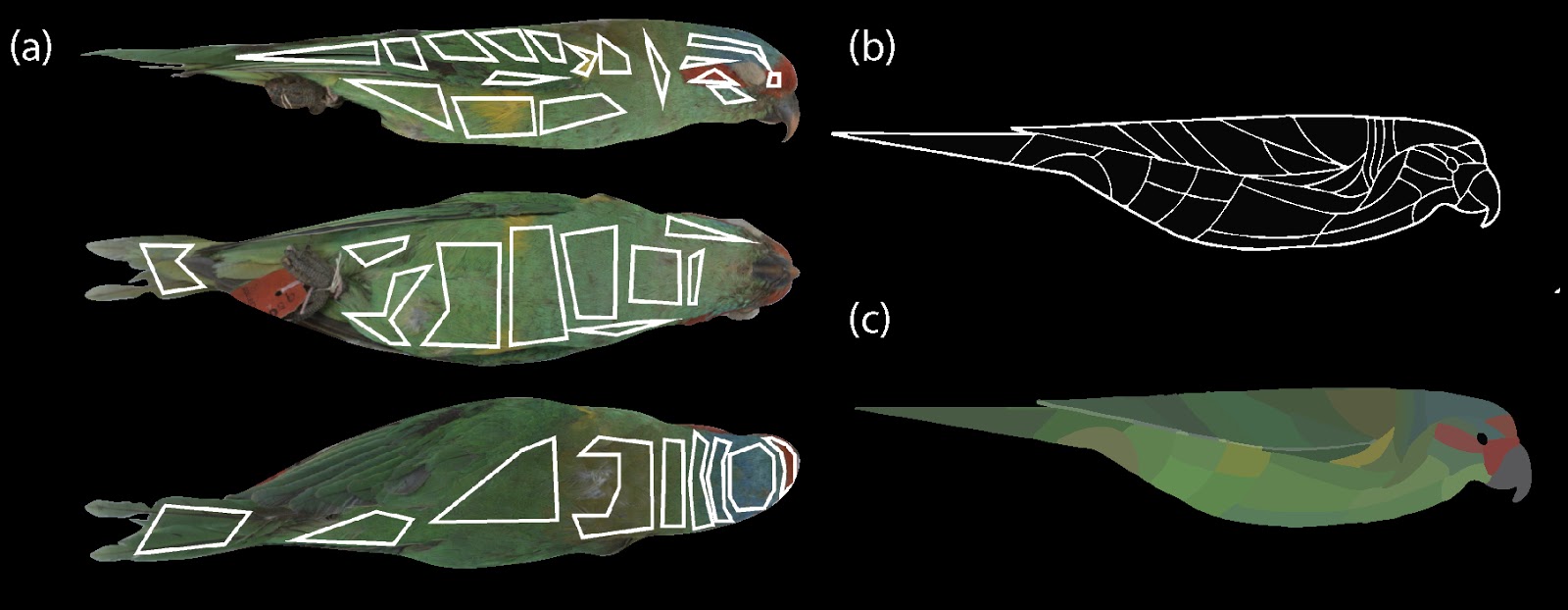


Figure S5: Example of sampling patches from an example photo (a). Patch sampling scheme was adapted from McKay (2013), while examining plates and skins to adequately capture full 3D bird variance. Patches were selected from three views.


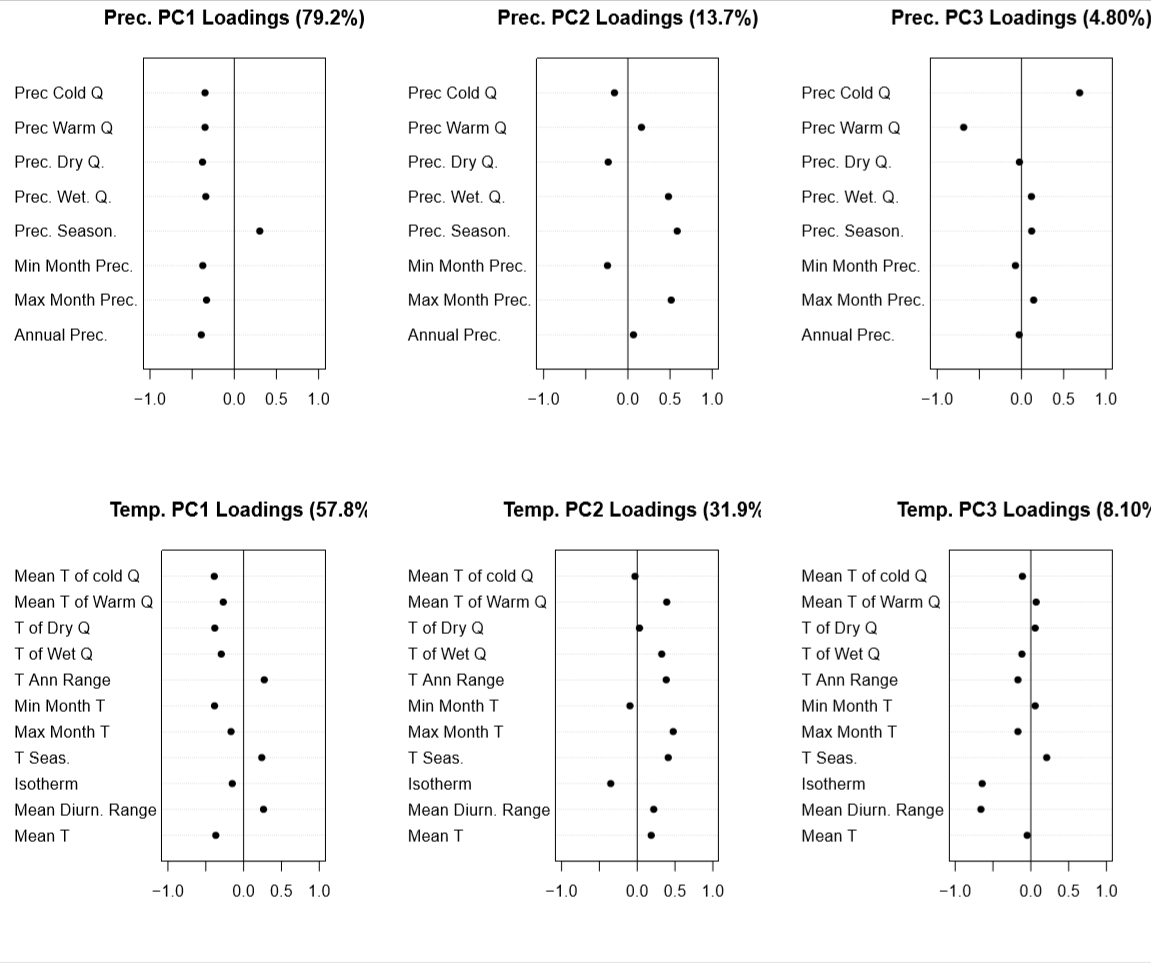


Figure S6: Principal component weights for precipitation and temperature. These are the principal components which were used for PGLS models. PC1 of Temperature explained 57.8% of variation in bioclim variables 1-11, and reflected temperature seasonality at high values and high mean temperature at low values. PC2 and PC3 of Temperature explained 31.9% and 8.10% of the variation and reflected variation along axes of mean and maximum quarterly temperatures and isothermality respectively. PC1 of Precipitation explained 79.2% of the variance in precipitation variables and mainly described variation between low and high mean seasonal precipitation. PCs 2 and 3 of Precipitation explained 13.7% and 4.80% of the variance in precipitation variables and best described variation in seasonality and precipitation in the coldest and warmest quarters respectively.


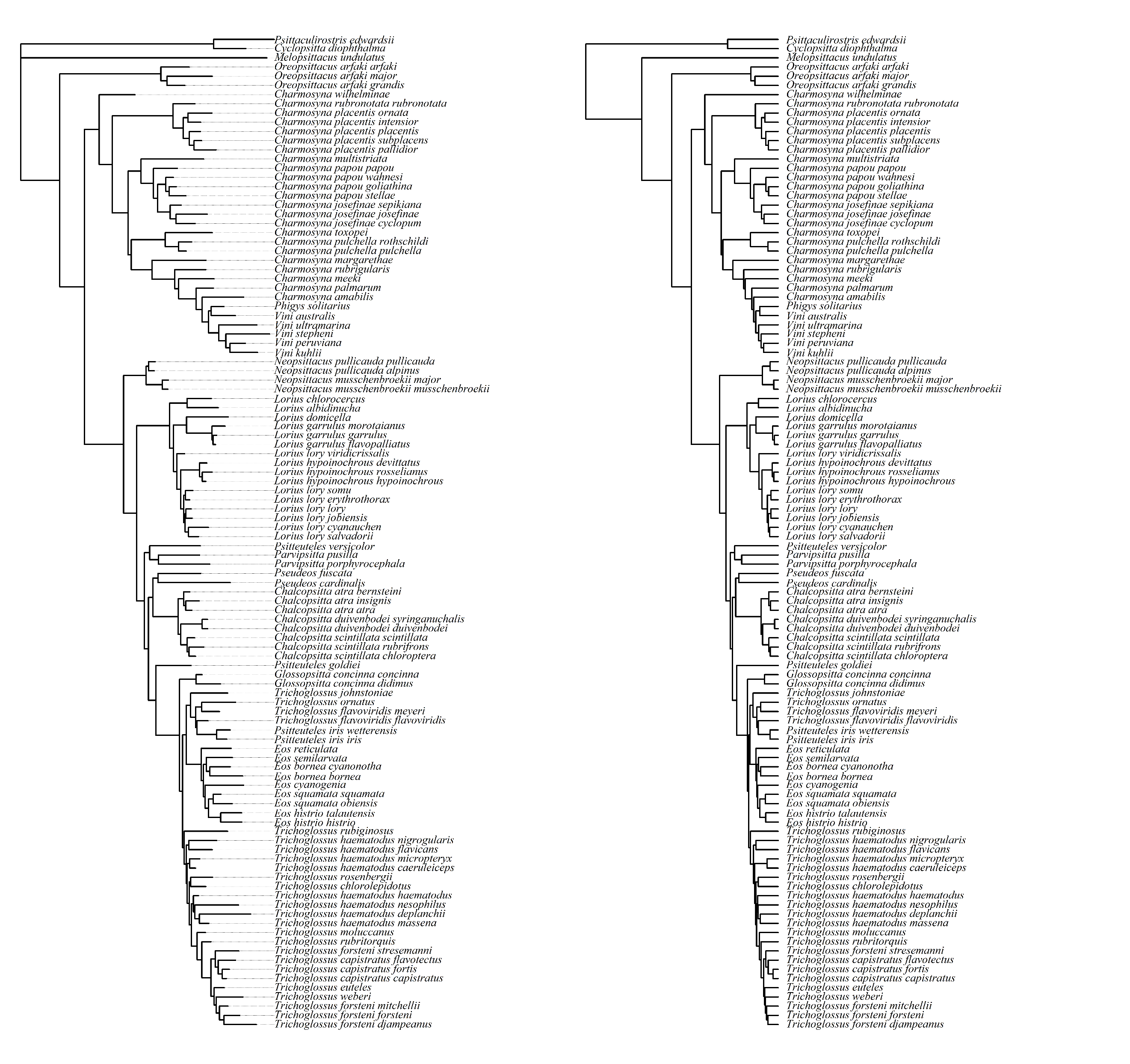


Figure S7: Comparison between uncalibrated (Supplementary Fig. S11, Smith et. al., 2019) and time-calibrated tree.
