# Supplementary figures and images for: Macroevolutionary bursts and constraints generate a rainbow in a clade of tropical birds
Source: BMC Evol Biol. 2020 Feb 24;20:32. doi: 10.1186/s12862-020-1577-y (PMC7041239; doi:10.1186/s12862-020-1577-y)

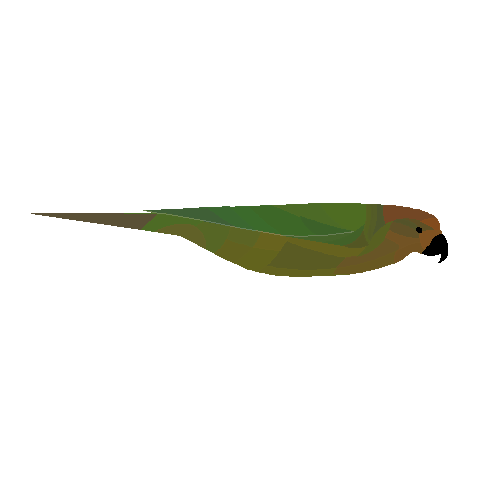

Supplement: Supplementary file 2 — Additional file 2: Electronic Supplement: Root to Lorius Animation. An animation between ancestral reconstructions at each node from root of all lorikeets to Lorius. [file 12862_2020_1577_MOESM2_ESM.gif]
